# Products of lipid, protein and RNA oxidation as signals and regulators of gene expression in plants

**DOI:** 10.3389/fpls.2015.00405

**Published:** 2015-06-02

**Authors:** Jagna Chmielowska-Bąk, Karolina Izbiańska, Joanna Deckert

**Affiliations:** Department of Plant Ecophysiology, Institute of Experimental Biology, Faculty of Biology, Adam Mickiewicz University, Poznań, Poland

**Keywords:** reactive oxygen species, signaling, oxidation, oxylipins, oxidized proteins, oxidized RNA

## Abstract

Reactive oxygen species (ROS) are engaged in several processes essential for normal cell functioning, such as differentiation, anti-microbial defense, stimulus sensing and signaling. Interestingly, recent studies imply that cellular signal transduction and gene regulation are mediated not only directly by ROS but also by the molecules derived from ROS-mediated oxidation. Lipid peroxidation leads to non-enzymatic formation of oxylipins. These molecules were shown to modulate expression of signaling associated genes including genes encoding phosphatases, kinases and transcription factors. Oxidized peptides derived from protein oxidation might be engaged in organelle-specific ROS signaling. In turn, oxidation of particular mRNAs leads to decrease in the level of encoded proteins and thus, contributes to the post-transcriptional regulation of gene expression. Present mini review summarizes latest findings concerning involvement of products of lipid, protein and RNA oxidation in signal transduction and gene regulation.

## Introduction

It is well established that reactive oxygen species (ROS) are engaged in the cellular signal transduction network (Neill et al., 2002; Ślesak et al., 2007; Wrzaczek et al., 2013). In the case of plants they have been shown to mediate morphogenesis, stomatal closure, gravitropism, programmed cell death (PCD), and responses to stress factors, including systematic acquired resistance (SAR) and systematic acquired acclimation (SAA; Neill et al., 2002; Ślesak et al., 2007). In recent years progress has been made in understanding the molecular language associated with ROS-mediated signal transduction. Changes in ROS level affect oxidative status of redox-sensitive signal regulators such as ascorbate, glutathione and thioredoxins, leading to modulation of metabolism and gene expression. For example, shift of ascorbate/dehydroascorbate ratio toward more oxidized state was shown to inhibit cell cycle (Kopczewski and Kuźniak, 2013). In turn, glutathione and thioredoxins are major players in protein thiol switching—reversible modification of proteins causing alterations in their conformation and functioning. Thiol switches were shown to regulate various cellular processes including translation, transcription and signaling (Brandes et al., 2009; Dietz and Hell, 2015). Changes in cellular redox status affects disulfide bonds in proteins associated with transcription, leading to the alterations in their polymerization and DNA binding capacity (Chi et al., 2013). Proteins subjected to the described oxidative-dependent regulation include NPR1 transcription co-activator and TGA9, RAP2.4a, and R2R3 transcription factors (Wrzaczek et al., 2013). ROS influence signal transduction pathways also indirectly through interaction with other signaling elements, such as nitric oxide, calcium ions and plant hormones (Neill et al., 2002; Chi et al., 2013). Direct and indirect ROS action modulates gene expression. The global *Arabidopsis* transcriptome analysis has revealed that application of H_2_O_2_ causes changes in the expression of approximately 1% of genes (Desikan et al., 2001).

**FIGURE 1:**
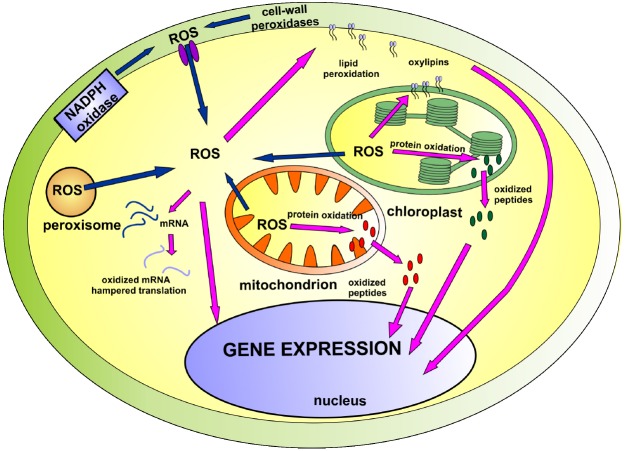
**The involvement of ROS and products of ROS-dependent oxidation in cellular signaling network.** The main sources of ROS (marked with blue arrows) include mitochondria, chloroplast, NADPH oxidase and cell-wall peroxidases. Accumulated ROS participate in cellular signaling (marked with pink arrows) directly or indirectly through derivatives of lipid, protein and mRNA oxidation. Detailed description can be found in the text.

Lately, new players have emerged on the ROS signaling arena. It has been suggested that oxidation of cellular compounds leads to the formation of new signaling molecules: non-enzymatically formed oxylipins, peptides derived from ROS-dependent protein degradation and oxidatively modified mRNAs. The present mini review summarizes their role in cellular signaling and gene regulation (illustrated on Figure 1).

## Lipid Peroxidation Products

Oxygenation of polyunsaturated fatty acids leads to the production of oxylipins *via* enzymatic or non-enzymatic pathways. The study of plant lipid peroxidation products, thus far has been focused mainly on the enzymatically synthesized jasmonate family of signaling molecules, and their roles in the regulation of developmental and defense-related processes. Importantly, it should be emphasized that the enzymatic process, which is generally seen as beneficial for survival, generates relatively small numbers of lipid regulators. Recent evidence indicates that a number of biological active oxylipins are generated in a non-enzymatic pathway, initiated by enhanced formation of ROS, mainly hydroxyl radical and superoxide anion. Moreover, a growing body of evidence suggests that some non-enzymatically formed oxylipins, such as phytoprostanes (PP), may also function as endogenous mediators that regulate various signal transduction pathways (Thoma et al., 2003; Loeffler et al., 2005; Grun et al., 2007). Transcriptome analysis have revealed that in *Arabidopsis* and tomato plants A1- and B1-type phytoprostanes (PPA1 and PPB1) induce a variety of genes associated with secondary metabolism, stress responses and cellular detoxification. Importantly, the induced enzymes included glutathione-S-transferase (GST) and cytochrome P450 enzymes are engaged in inactivation of toxic electrophiles, which are also formed in a result of lipid peroxidation. Therefore, it has been suggested that phytoprostanes are not only markers of oxidative injury, but may also act as ROS scavengers (Thoma et al., 2003; Loeffler et al., 2005; Mueller et al., 2008). Moreover, it has been shown that 24% of the genes in *Arabidopsis* up-regulated by PP are related to signal transduction, including transcription factors, phosphatases and kinases (Mueller et al., 2008). Interestingly, Mueller et al. (2008) also reported that approximately 50% of the PPA1-induced genes in both *Arabidopsis* cell culture and whole-plant experiments contain a TGA motif (TGACG) in their promoters. These are putative binding sites for basic/leucine zipper transcription factors of the TGA family. Subsequent analysis revealed that 60% of all genes induced by PPA1 could not be induced in *Arabidopsis* mutants deficient in class II TGA transcriptions factors—*tga2*, *tga5*, and *tga6* mutants (Mueller et al., 2008). This class of transcription factors are known to be involved in plant xenobiotic signaling (Behringer et al., 2011). In response to a variety of xenobiotics, they recruit the SCARECROW-LIKE 14 (SCL14) transcriptions coactivator to promoters with TGA motifs and induce the expression of detoxification genes. The fact that *Cyp81D11* (*At3g28740*) gene encoding a putative cytochrome P450 monooxygenase, is induced by both PPA_1_ and TGA/SCL14 complex indicates that there is a link between oxylipin signaling and TGA-mediated gene regulation in response to xenobiotic metabolism (Fode et al., 2008; Mueller et al., 2008). Although the substrate of *CYP81D11* is not known, its presumed function as a monooxygenase and its expression pattern suggest a role in plant detoxification processes (Fode et al., 2008; Mueller et al., 2008).

## Oxidized Proteins and Peptides

Gadjev et al. (2006) conducted analysis of data on changes in *Arabidopsis* transcriptome in response to oxidative stress of various origins. Four out of nine analyzed microarray studies were conducted on plants treated with ROS-generating agents: ozone leading to the formation of H_2_O_2_ and O_2_^–^ in apoplast, methyl viologen (MV)—redox-cycling herbicide generating O_2_^–^ in chloroplasts and mitochondria, 3-aminotriazole (AT)—herbicide causing H_2_O_2_ accumulation in peroxisomes and AAL—toxin leading to drastic increase of cellular H_2_O_2_ level. The rest of the experiments were preformed on either *flu* mutant characterized by rapid accumulation of ^1^O_2_ in chloroplasts after transfer from dark to light conditions or transgenic plants with alerted antioxidant systems in specific cell compartments including chloroplasts, mitochondria, cytoplasm and peroxisomes (Gadjev et al., 2006; and references therein). The results of the study indicated that, in addition to hallmark transcripts induced by general oxidative stress, a number of genes respond specifically only to a particular ROS specie or even to ROS originating from a specific cell compartment. It is worth mentioning that these specific ROS signatures can be now identified in *Arabidopsis* transcriptome by a bioinformatic tool—ROSMETER. The software estimates correlation between microarray data of interest and indices compiled from available transcriptomic data on *Arabidopsis* plants undergoing oxidative stress of different type and origin (Rosenwasser et al., 2013). Although, it seems that plants possess a sensory system which enables them to distinguish between ROS produced in specific organelles, the exact mechanism of such system is still unknown. It has been proposed that the required information is transmitted through H_2_O_2_ waves differing in frequency and amplitude, as has been observed in the case of Ca^2+^ (Mittler et al., 2011). However, Ca^2+^-signaling requires the maintenance of low Ca^2+^ basal level by efficient removal and storing in relaying stations, such as vacuoles and endoplasmic reticulum (ER). In contrast, the level of H_2_O_2_ in plant cells is relatively high. The steady state concentration of H_2_O_2_ has been estimated in the range between 60 μM and 2 mM depending on plant species. However, it is possible that such wide range might result from technical difficulties in ROS quantification (Neill et al., 2002; and references therein; Vestergaard et al., 2012). There is also no evidence that ROS can be stored in any relaying station. A study based on extensive mathematical modeling demonstrated that, taking into account the above-mentioned facts, the transmission of a signal by diffusing H_2_O_2_ is unlikely (Vestergaard et al., 2012). Another possibility is that the generated ROS activate other, more specific signaling elements (Mittler et al., 2011). Møller and Sweetlove (2010) have proposed that functions of such specific signaling elements could be performed by peptides derived from ROS-dependent degradation of proteins. The peptides derived from protein oxidation are capable of transmitting several pieces of information. Firstly, the origin of degraded protein gives information about the localization of oxidative stress. Secondly, the number of derived peptides reflects the intensity of oxidative stress. Lastly, the modification of determined amino acid residues is a signature of the type of over-produced ROS specie (Møller and Sweetlove, 2010). Interestingly, it has been already demonstrated that peptides derived from protein degradation exhibit signaling functions. In *C. elegans* response to unfolded protein stress in mitochondria, involving induction of chaperone genes, requires re-localization of the ZC376.7 protein from cytoplasm to nucleus. Translocation of this transcription factor is dependent on the activity of two mitochondria-localized proteins—Clp protease and HAF-1 transporter. The authors propose that unfolded/misfolded proteins are degraded by Clp protease, and the derived peptides are transported by HAF-1 to cytoplasm, where they interact with ZC376.7, leading to its translocation to the nucleus (Haynes et al., 2010). However, to our best knowledge, so far there are no studies fully supporting the idea that also peptides formed as a result of protein oxidation act as signaling elements. Providing experimental evidence for involvement of such peptides in organelle-specific ROS signaling is an interesting future research challenge.

## Oxidatively Modified RNA

Although most of the research concerning nucleic acid oxidation is concentrated on DNA, several reports imply that RNA is more susceptible to this process (Hofer et al., 2006; Shan et al., 2007; Liu et al., 2012). Importantly, the accumulation of oxidized RNA has been shown to be associated with several disorders, such as Parkinson’s and Alzheimer’s diseases, amyotrophic lateral sclerosis (ALS), dementia with Lewy bodies, schizophrenia, diabetes and hemochromatosis (Nunomura et al., 2004; Poulsen et al., 2012; Jorgensen et al., 2013 and reference therein). Immunoprecipitation with monoclonal antibody 15A3 recognizing markers of nucleic acid oxidation, 8-oxo-7,8-hydroguanosine (8OHG) and 8-oxo-7,8-dihydroguanine (8OHdG), enabled identification of mRNAs highly oxidized in postmortem isolated brain tissues of patients suffering from Alzheimer’s disease. Interestingly, oxidation did not occur in abundant transcripts encoding for example β-actin. Instead the most prominent oxidation was observed in the case of a specific set of mRNAs associated with signaling, protein transport, regulation of gene expression, response to oxidative stress and metabolism. Thus, the authors conclude that mRNA oxidation is not a random process but targets specific transcripts (Shan et al., 2003). Similar results were obtained in motor neurons of transgenic mice expressing familial ALS-linked mutation in *superoxide dismutase* gene. In three independent microarray analysis performed in identical experimental conditions oxidation affected same RNA species. Interestingly some of the highly oxidized mRNAs were associated with ASL pathogenesis (e.g., superoxide dismutase, amyloid beta precursor-like protein, prion protein) and cellular signaling (e.g., calmodulin, mitogen-activated protein kinases, transcription factors; Chang et al., 2008). Targeted mRNA oxidation has been also observed in plants. Increased levels of 8-oxo-7,8-dihydroguanosine (8-OHG), a marker of RNA oxidation, were noted in sunflowers seeds. The observed oxidation was not a random process—reproducible microarray analysis showed that only a specific set of mRNAs has been oxidatively modified (Bazin et al., 2011). Thus far it is not clear what determines the susceptibility of mRNA to oxidation. However, the possibilities that its sensitivity is dependent on the abundance of specific mRNA species, the frequency of guanine in its sequence, or the occurrence of any specific motif have been excluded (Chang et al., 2008; Bazin et al., 2011). Importantly accumulation of oxidized transcripts causes ribosome stalling leading to the slowing down of translation (Shan et al., 2007; Simms et al., 2014), which can explain frequently observed reduction in the level of encoded proteins (Shan et al., 2003, 2007; Tanaka et al., 2006; Chang et al., 2008). Thus, targeted RNA oxidation leading to the diminished expression of definite proteins might constitute a newly discovered mechanism of post-transcriptional gene expression regulation. Moreover, as highly oxidized transcripts encode inter alia signaling elements, this process likely affects cellular signaling network (Shan et al., 2003; Chang et al., 2008; Bazin et al., 2011).

### Conflict of Interest Statement

The authors declare that the research was conducted in the absence of any commercial or financial relationships that could be construed as a potential conflict of interest.
